# Anaphylactic shock during cement implantation of a total hip arthroplasty in a patient with underlying mastocytosis: case report of a rare intraoperative complication

**DOI:** 10.1186/s13037-016-0113-x

**Published:** 2016-11-05

**Authors:** Anita ten Hagen, Pieter Doldersum, Tom van Raaij

**Affiliations:** 1 Department of Anesthesiology, Martini Hospital Groningen, Van Swietenplein 1, 9728 NT Groningen, The Netherlands; 2 Department of Orthopaedic Surgery, Martini Hospital Groningen, van Swietenplein 1, 9728 NT Groningen, The Netherlands

**Keywords:** Mastocytosis, Bone cement, Implantation syndrome, Anaphylaxis, Hip surgery, Case report

## Abstract

**Background:**

Cemented total hip arthroplasty (THA) is a safe and common procedure. In rare cases life threatening bone cement implantation syndrome (BCIS) may occur, which is commonly caused by pulmonary embolism (PE).

**Case presentation:**

We describe the rare case of a 70-year old patient who underwent an elective total hip replacement. Before surgery he was diagnosed with underlying systemic indolent mastocytosis, a rare pathological disorder that may result in anaphylaxis after massive systemic mast cell activation. Triggers may be IgE-mediated, direct mast cell activation, or unclear. Some patients may be at risk for severe non IgE-mediated reactions, such as those experienced with nonsteroidal anti-inflammatory drugs, or with perioperative muscle relaxants. During cementing of the acetabular component, our patient developed acute hypotension (blood pressure dropped from 90/50 to 60/40 mmHg, and saturation dropped from 95 to 80 %). The differential diagnosis of acute PE was excluded (no signs of breathing abnormalities during physical examination, normal arterial blood sample, and no electrocardiography or cardiac ultrasound abnormalities). The patient was diagnosed with acute anaphylactic shock, which was successfully managed by 100 % oxygen administration, rapid fluid induction, and vasoconstrictive drug therapy. He recovered hemodynamically within 15 min, did not lose consciousness, and did not develop angioedema or an urticarial rash. Forty-five minutes after onset of the symptoms, the surgical procedure was completed after inserting a press fitted uncemented femoral stem component. The patient was transported to the Intensive Care Unit (ICU) for optimal monitoring. Our patient had an uneventful recovery. Within six hours after surgery he started to ambulate following our standard fast-track rehabilitation regime. Post-operative day one he was discharged to the specialized Orthopedic Department, and after five hospital days discharged to his home. Twelve months after THA surgery our patient was satisfied with an optimal functional status of his hip joint replacement.

**Conclusion:**

The differential diagnosis of anaphylactic shock must be taken into consideration in patients with acute hypotension during cementing of total hip arthroplasty components. Patients with underlying mastocytosis are at particular risk of this potential life-threatening intra-operative complication. This rare entity should be taken into consideration during the pre-operative risk stratification and shared decision-making process for elective cemented joint replacement.

## Background

BCIS is characterized by a number of clinical features that may include hypoxia, hypotension, cardiac arrhythmias, increased pulmonary vascular resistance, and cardiac arrest. BCIS is most commonly associated with hip arthroplasty, and usually occurs at one of the five stages in the surgical procedure; femoral reaming, acetabular or femoral cement implantation, insertion of the prosthesis or joint reduction [1]. High incidence with severe reactions has been reported in cemented hemi-arthroplasty after a proximal femur fracture. In elective THA surgery the incidence has been much lower, but still the rate of intraoperative mortality is about 0.1 % [2]. Diverse mechanisms have been postulated as causing BCIS [1]. An anaphylactoid mechanism as a major factor contributing to the development of BCIS remains disputed. Earlier, Tryba et al. showed increased levels of plasma histamine in elderly patients with hip fractures after the implantation of acrylic bone cement into the femur [3]. Mitsuhata et al. concluded that methylmethacrylate bone cement does not release histamine during total hip replacement surgery [4]. A randomized trial in 50 patients receiving cemented hip arthroplasty could not demonstrate a prophylactic potential for histamine-receptor blocking agents in BCIS [5]. Since then, many studies have suggested that pulmonary embolization is the main cause in BCIS [1]. Mastocytosis is a rare pathological disorder that may result in anaphylaxis after massive systemic mast cell activation. Triggers may be IgE-mediated, direct mast cell activation, or unclear. IgE-mediated anaphylaxis to drugs and foods are not well studied [6]. We describe a patient diagnosed with systemic indolent mastocytosis who most likely developed an anaphylactoid BCIS response after acetabular component cementation in elective total hip surgery.

## Case presentation

A-70-year-old man with symptomatic osteoarthritis of the right hip, weighing 79 kg, tall 177 cm was presenting for a primary THA. He was recently diagnosed with systemic indolent mastocytosis with multiple sclerotic and osteolytic laesions. History showed no allergic reactions in the past. In 1998 cannulated hip screws were used to treat a left proximal transcervical femur fracture successfully. Multiple insufficiency vertebra fractures were treated non-operative in 2015. A multimodal oral analgesic regime consisting of paracetamol 1 g, naproxen 500 mg and gabapentin 600 mg was administered 1–2 h pre-operatively. On the pre-anesthetic care unit the blood pressure was 150/95 mmHg with a saturation of 95 % and 11,85 mg Dexamethasone and Cefazoline 2000 mg soluted in 20 ml Sodiumchloride (NaCl) 0.9 % was administered intravenously. In the operating room spinal anesthesia with 7.5 mg bupivacaine 0,5 % plain via the L2/3 vertebral interspace with a standardized intraoperative regime for fluid administration, consisting of 0,9 % saline (500 ml) was given. After spinal anesthesia the patient received sedation with propofol TCI 0,8 ug/ml (0,1–2.0 ug/ml). To reduce blood loss, tranexamic acid 1000 mg was given at the beginning of surgery. To reduce nausea and vomiting ondansteron 4 mg IV is added in this multimodal regimen. To reduce chronic pain, ketamine 15 mg IV is titrated after starting the sedation. The blood pressure was 120/70 mmHg after spinal anesthesia and starting the sedation. Three liter of Oxygen was added during sedation. Thirty minutes after induction the orthopedic surgeon inserted a cemented acetabular component. Blood pressure dropped from 90/50 to 60/40 mmHg. Saturation dropped from 95 to 80 %. Oxygen 100 % with a Ventimask was administered and rapid infusion of NaCl 0.9 % 1000 ml as well as Phenylephrine 4 × 100 ug, phenypephrine 2 × 200 ug and 10 mg ephedrine was given intravenously. Phenylephrine infusion was started and replaced by Noradrenaline 5 mg/50 ml NaCl 0.9 % 2.0–9.0 ml/h. The breath sounds remained normal, and no angioedema, urticaria or rash occurred. All the time, the patient was conscious and able to respond questions. With rapid infusion and finally Noradrenaline he recovered hemodynamically within 15 min, blood pressure 100/50 mmHg with 95 % saturation. Forty-five minutes after onset of the symptoms, the surgical procedure was completed after inserting a press fitted uncemented femoral stem component. The patient was transported to the Intensive Care Unit (ICU) for optimal postoperative treatment and monitoring. Biochemistry showed stable hemoglobin levels (8.3 mmol/l on ICU arrival and 8.4 mmol/l after 4 h), elevated white blood cell count (19.3 × 10^9^/l), normal levels of electrolytes, liver enzymes, creatine kinase and troponin, and an arterial blood sample of pH 7.36 pCO2 6.1 kPa; pO2 14.4 kPa; HCO3 26 mmol/l; Base excess 0.4 mmol/l; Fys. O2 sat 98.7 %; FiO2 30 %; lactic acid 1.0 mmol/l. Electrocardiography (on ICU arrival and after 24 h) remained normal, and echocardiography showed no left or right ventricular disfunctioning or elevation of the right atrial pressure. Within six hours after surgery the patient started to ambulate following our standard fast-track rehabilitation regime. Post-operative day one he was discharged according strict functional criteria to the specialized Orthopedic Department. After 5 hospital days the patient was discharged to his home.

## Discussion

BCIS has been observed in prosthetic hip surgery for many years, and is still not fully understood. A severity classification has been proposed; grade 1: moderate hypoxia (SpO2,94 %) or hypotension [fall in systolic blood pressure (SBP) .20 %], grade 2: severe hypoxia (SpO2 ,88 %) or hypotension (fall in SBP .40 %) or unexpected loss of consciousness, and grade 3: cardiovascular collapse requiring cardiopulmonary resuscitation [1]. In our case a grade 2 BCIS occurred after cementing the acetabular component; systemic blood pressure drop more than 30 % and severe hypoxia (saturation 80 %). The patient recovered in 15 min after rapid fluid induction, 100 % oxygen administration, and vasoconstrictive drug therapy. He did not lose consciousness, did not develop angioedema or an urticarial rash, and remained hemodynamic stable with blood loss not more than expected after elective hip surgery. All our eligible patients receive cemented acetabular components during total hip surgery. Nationwide registry shows better performance of cemented cups over uncemented cups [7]. Also less damage to the acetabular host bone with a cemented component during future revision surgery is to be expected [8]. Acetabular pressure rises after cup placement, especially during cementation, and an average of 375 mmHg has been reported [9]. As a consequence extrusion of bone marrow content with diffuse microembolism of the lungs may occur. In our case pulmonary fat embolism was excluded; there were no signs of breathing abnormalities during physical examination, only a short period of hypoxia occurred, and no electrocardiography or cardiac ultrasound abnormalities were encountered. Normovolemic hypotension without angioedema or rash may also be caused by an anaphylactoid mechanism observed in mastocytosis [6]. Recently our patient was diagnosed with systemic indolent mastocytosis through an iliac crest biopsy. Anaphylaxis in mastocytosis can be either IgE-mediated or non IgE-mediated, such as those experienced with nonsteroidal anti-inflammatory drugs or muscle relaxants. In our hospital all primary THA patients undergo a standardized anesthesia, analgesia and perioperative program to enhance recovery. Low dose bupivacaine and a multimodal opioid-sparing regimen facilitate early mobilization and allow rehabilitation to be initiated a few hours postoperatively. Our patient received intravenous nonsteroidal anti-inflammatory drugs at least one hour preoperatively, and no muscle relaxants were given. The acute response after cement application suggests a reaction on polymethylmethacrylate (PMMA) bone cement. Earlier, PMMA mediated histamine release with increased plasma histamine concentrations after bone cement implantation in the femoral shaft has been described [4]. More recent hypersensitivity to PMAA following joint replacement has also been reported [10, 11]. Krüger et al. described a patient with mastocytosis who underwent kyphoplasty for multiple osteoporotic vertebral fractures using radiopaque calcium phosphate bone cement under antihistamine and prednisolone coverage [12]. Arterial hypotension was encountered that transiently required vasopressor support. The critical threshold of intramedullary pressure that causes fat embolism is unclear, but pressure peaks as great as 300 mmHg were found to produce significant bone marrow release [13]. Intravertebral pressure during kyphoplasty, however, is substantially lower, and remains well below 300 mmHg [14]. A pressure-induced mast cell degranulation mechanism seems more suitable. Although rare an allergic reaction to calcium phosphate cement has been noted in skull reconstruction [15]. Even severe hypoxia with ventricular failure as a sign of possible aggregate anaphylaxis has been seen after formation of intravenous calcium phosphate precipitant [16]. Therefore, it seems more likely that Krügers’ observation and our case highlight the capacity of bone cement to trigger a disproportionate display of mast cell activity in mastocytosis patients with a BCIS response as a result. The use of corticosteroids has been advised to prevent anaphylaxis in patient with mastocytosis. In both patients corticosteroids were administered before intervention, which did not prevent BCIS unfortunately.

## Conclusion

Our case suggests a disproportionate response to PMMA bone cement, which lead to anaphylactic shock. Patients with underlying mastocytosis are at particular risk of this potential life-threatening intra-operative complication. This rare entity should be taken into consideration during the preoperative risk stratification and shared decision-making process for elective cemented joint replacement.

## Abbreviations

BCIS: Bone cement implantation syndrome
ICU: Intensive Care unit
NaCl: Sodiumchloride
PE: Pulmonary embolism
PMMA: Polymethylmethacrylate
SBP: Systemic blood pressure
THA: Total hip arthroplasty
